# Lattice Dynamics of Cu_2_ZnSn(S_*x*_,Se_1–*x*_)_4_ Kesterite Thin-Film Solar Cells Studied by Nuclear Inelastic Scattering

**DOI:** 10.1021/acs.jpcc.4c03689

**Published:** 2024-10-07

**Authors:** Raju Edla, David Nowak, Dirk Hauschild, Ilya Sergueev, Devendra Pareek, Levent Gütay, Clemens Heske, Lothar Weinhardt, Svetoslav Stankov

**Affiliations:** † Institute for Photon Science and Synchrotron Radiation (IPS), Karlsruhe Institute of Technology (KIT), Karlsruhe 76131, Germany; ‡ Ultrafast Nanoscale Dynamics, Institute of Physics, Carl von Ossietzky University of Oldenburg, Oldenburg 114-118 26129, Germany; § Institute for Chemical Technology and Polymer Chemistry (ITCP), Karlsruhe Institute of Technology (KIT), Karlsruhe 76131, Germany; ∥ Department of Chemistry and Biochemistry, University of Nevada, Las Vegas (UNLV), Las Vegas, Nevada NV 89154, United States; ⊥ Deutsches Elektronen-Synchrotron DESY, Hamburg 22607, Germany; # Laboratory for Applications of Synchrotron Radiation (LAS), Karlsruhe Institute of Technology (KIT), Karlsruhe 76131, Germany

## Abstract

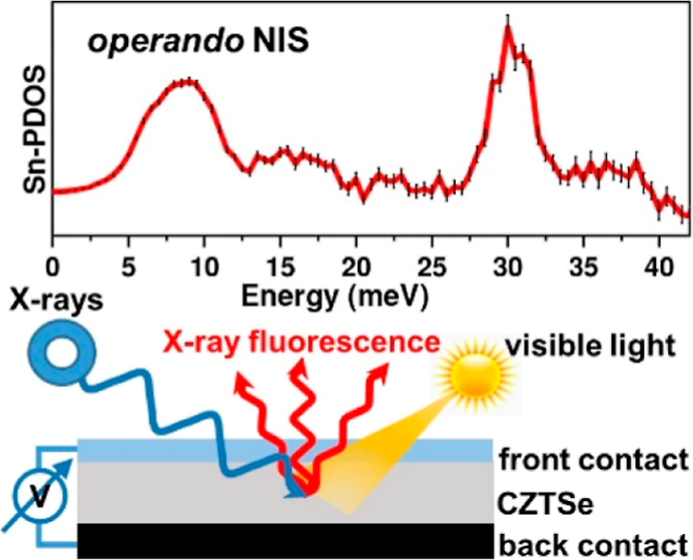

Phonons play a crucial role in thermalization and non-radiative recombination losses in semiconductors, impacting the power conversion efficiency of solar cells. To shed light on the lattice dynamics in Cu_2_ZnSn(S_*x*_,Se_1–*x*_)_4_ (CZTSSe) thin-film solar cells and validate the extensive number of theoretical studies, we determine the ^119^Sn-partial phonon density of states (Sn-PDOS) by nuclear inelastic X-ray scattering. CZTSSe-based devices, one with near-stoichiometric and two with off-stoichiometric compositions, are investigated, and the results are correlated with the corresponding power conversion efficiencies (PCEs) of 3.2, 7.6, and 10.6%, respectively. Compared to the near-stoichiometric cell, the main Sn-PDOS peak of the off-stoichiometric cells broadens and slightly shifts to higher energy; this effect is correlated with the type and concentration of the characteristic defects in the studied samples. Furthermore, the Sn-PDOS of the 10.6% device is also obtained under *operando* (maximum power point) and open-circuit conditions. A comparison of the Sn-PDOS before and after the *operando* measurements suggests structural changes, likely due to the formation of metastable defects. In agreement with the theoretical studies, the Sn-PDOS of the CZTSSe absorber shows additional peaks compared to CZTSe attributed to coupling of Sn to the vibrations of Se and S atoms. This work paves the way for a further understanding of the lattice dynamics and subsequent enhancement of the PCEs of thin-film solar cells as well as other applied materials and devices containing elements that are Mössbauer-active and hence suitable for nuclear inelastic scattering.

## 1 Introduction

Efficient solar energy harvesting, via photovoltaic (PV) or photoelectrochemical water splitting (PEC) technologies, has the potential to help meet the continuously growing energy demand by providing green renewable energy.^1^ Intense research efforts in the past decades have been devoted to optimizing the existing and discovering new materials and devices, which resulted in a dramatic increase of power conversion efficiencies (PCEs)^2^ across many different technologies. Devices with kesterite-based absorbers [Cu_2_ZnSn(S_*x*_,Se_1–*x*_)_4_, (CZTSSe)], being an earth-abundant, highly absorbing p-type direct band gap semiconductor with an adjustable band gap^3^ between ∼1.0 and 1.5 eV, have attracted a growing attention^4^ for low-cost rigid and flexible thin-film solar cells^5−11^ and recently as photocathodes for efficient solar hydrogen production.^12−14^

CZTSSe has been studied by various characterization techniques to improve our understanding of its structural, chemical, electronic, and thermodynamic properties.^11,15−19^ It was recognized that kesterite solar cells with off-stoichiometric composition, i.e., Cu-poor and Zn-rich, give the best power conversion efficiency (PCE), with a current record of 14.9%.^2^ However, this is significantly lower than expected—the theoretical detailed balance limit^20^ for a single-junction device at ∼1.0 eV would be 28%.^15,21^ Other commercially available thin-film solar-cell devices [e.g., with Cu(In,Ga)Se_2_ or CdTe absorbers] show significantly higher efficiencies.^2^ The comparatively low efficiency of kesterite solar cells is mainly attributed to a large open-circuit voltage (*V*_OC_) deficit, which could be due to the presence of defects, secondary phases, and/or the spontaneous transition from a kesterite to a stannite structure.^15,22,23^ The off-stoichiometric configuration of the kesterite structure introduces point defects such as vacancies, anti-sites, and interstitials, which significantly influence its optoelectronic properties.^15,24,25^ An optimized concentration of these defects is expected to be beneficial for enhancing the kesterite solar cell efficiency.^26^

Thermal lattice excitations (phonons) play a crucial role in the thermalization and non-radiative recombination of the photoexcited charge carriers in a PV solar cell, which constitutes a fundamental limitation for achieving the radiative-limited PCEs given by the detailed balance limit.^20,21^ The thermalization mechanism involves interactions of the hot electrons with longitudinal optical (LO) phonons, described by the Fröhlich coupling.^27^ The emitted LO phonons quickly decay to acoustic phonons by Klemens^28^ and/or Ridley^29^ processes. Acoustic phonons, being the main heat carriers in semiconductors and insulators, exhibit large group velocities and can propagate across interfaces, which leads to an irretrievable energy loss and hence lowers the PCE of the solar cells.^30^

Furthermore, the thermodynamic, elastic, and optical properties of a crystal are determined by the vibrational dynamics of the crystal lattice. A precise knowledge of the lattice dynamics is thus essential for understanding the properties of CZTSSe kesterites, including the unusually low thermal conductivity relative to other semiconductors,^31^ the ground-state crystal structure (kesterite, stannite, or a mixture of both),^32^ and thermal expansion and electron–phonon coupling.^33^

Phonon dispersion relations and element-specific phonon density of states (PDOS) of CZTSSe with various stoichiometries and crystal structures, as well as of different non-radiative recombination centers, have been extensively investigated by density functional theory (DFT).^16,32,34−37^ The impact of high-energy phonon states, electron–phonon coupling, and lattice anharmonicity on the PCEs have been pointed out.^36−38^ Despite comprehensive theoretical studies, experimental data on the phonon dispersions and the PDOS of real-world solar cells is missing.

The experimental determination of the lattice dynamics of solar cells under *operando* conditions (i.e., during device operation) remains a formidable challenge. Raman spectroscopy, standardly used for the structure characterization of the solar absorbers, probes only the Raman-active phonons at the center of the Brillouin zone, thus providing very limited information. Classical methods to measure the phonon dispersions in the entire Brillouin zone, such as inelastic neutron or X-ray scattering, are unfeasible not only due to the small sample volume but also because of the multielemental composition and multilayer structure of a thin-film solar cell. Nuclear inelastic scattering (NIS),^39,40^ on the other hand, is an element- and isotope-specific method, which provides the partial PDOS of Mössbauer-active isotopes such as ^119^Sn (natural abundance: 8.6%; average atomic mass of Sn: 118.71 u). The high penetration depth of X-rays with an energy of 23.88 keV, corresponding to the resonant transition^41^ in the nucleus of ^119^Sn, combined with the sensitivity of the method to thin films,^42^ renders this technique ideal for investigating the lattice dynamics of Sn-containing thin-film solar cells (and other devices) with a multilayered structure.

Here, we report NIS experiments on ^119^Sn under dark and illuminated conditions to determine the Sn-PDOS as well as thermodynamic and elastic properties of CZTSSe-based thin-film solar cells with different compositions and PCEs. The results reveal variations in the phonon frequency in relation to the stoichiometry variation (type and concentration of defects) and the solar cell efficiency. The formation of metastable defects is inferred from the *operando* NIS experiment of the device with the highest efficiency (10.6%). The experimentally obtained Sn-PDOS are in agreement with the results from the large body DFT calculations in the literature.^16,32,34−37^

## 2 Experimental Section

### 2.1 Preparation of the Kesterite Thin-Film Solar Cells

At the University of Oldenburg, samples were prepared by a two-step process. The first step was the sputter deposition of a Cu–Sn alloy and elemental Zn precursors on a Mo-coated soda-lime glass substrate at room temperature. In the second step, the deposited layers were annealed in a Se- and Sn-containing atmosphere in a tube furnace at 803 K for 20 min and naturally cooled to room temperature to obtain the CZTSe absorber with a thickness of approximately 2 μm. Afterward, to realize solar-cell devices, a buffer layer (∼50 nm chemical bath-deposited CdS) and a transparent front contact (75 nm i-ZnO and 500 nm Al:ZnO) were sputter-deposited on top of the absorbers. For the S4 absorber, an additional rapid annealing step under a H_2_S atmosphere was performed to exchange 25% of the Se by S. Further details on the deposition process can be found elsewhere.^10^ The compositions and achieved efficiencies of the cells are summarized in Table 1. The CZTSe absorber features a kesterite-like structure; the crystal lattice consists of alternating layers of Cu–Sn, Cu–Zn, and Cu–Sn bonds, separated by Se layers. A more detailed description of the structure can be found in refs and (32).

**Table 1:** Composition (Standard Deviation in Parentheses) of the Investigated Samples, Determined from Energy-Dispersive X-ray Spectroscopy (EDX) Measurements^a^

sample	efficiency	relative composition (at %)			ratio		
	%	Zn	Sn	Cu	Cu/(Zn+Sn)	Zn/Sn	S/(S+Se)
S1	3.20 (0.16)	26.16 (0.05)	26.10 (0.15)	47.74 (0.20)	0.91	1.00	0
S2	7.60 (0.31)	31.70 (0.66)	24.93 (0.32)	43.37 (0.38)	0.76	1.27	0
S3	10.60 (0.33)	33.89 (0.51)	23.63 (0.14)	42.47 (0.34)	0.74	1.43	0
S4	4.00 (0.77)	32.15(0.50)	24.95 (0.14)	42.90 (0.41)	0.75	1.29	0.25

a In sample S4, 25% of Se was replaced with S^10^.

### 2.2 Nuclear Inelastic Scattering Experiment

The NIS experiments at ^119^Sn nuclear resonance were conducted at the High Resolution Dynamics beamline P01^43^ at PETRA III (DESY, Hamburg) using X-rays with an energy of 23.88 keV. The instrumental resolution was 1.2 meV (full width at half-maximum, FWHM). The three CZTSe solar cells (S1–S3) and one CZTS_0.25_Se_0.75_ absorber sample (S4) were illuminated at a grazing angle of 0.5° with an X-ray beam of 1.0 × 0.045 mm^2^ (h × v). The high energy of the X-rays ensures that the entire depth of the CZTSSe layer (attenuation length of approximately 9 μm) is probed during the measurements. The measurements on all samples were conducted at 295 K. Additionally, the Sn-PDOS of sample S1 was obtained at 37 K using a helium-cooled continuous-flow cryostat.

The NIS spectra were measured in the range from −10 to +50 meV around the nuclear resonant energy by an avalanche photodiode positioned 1 mm above the thin-film solar-cell surface (Figure S1 and Supporting Information). The Sn-PDOS was obtained^44^ from the experimental NIS spectra in a quasi-harmonic approximation. Sample S3 was further investigated during illumination and operation at the maximum power point (MPP = 2.7 mW, *V* = 295 mV, *I* = −9 mA, referred to as “*operando*”; details about the experimental setup and calculation of the “MPP” are provided in the Supporting Information) and “open-circuit” conditions (*V*_OC_ = 350 mV) under irradiation with two halogen cold light sources (PHILIPS, *P* = 150 W, *T* = 3400 K). During these measurements, the distance between the device surface and the detector was increased to allow for efficient light irradiation, which reduced the count rate by about a factor of 3. In order to detect possible structural changes that could take place during device operation, the Sn-PDOS of sample S3 was obtained under dark conditions before and after the *operando* and open-circuit measurements. The temperature of the sample surfaces during the dark measurements was 295(1) K. During the *operando* and open-circuit experiments, the temperature of the solar-cell surface was measured by using a Lakeshore device with a Cernox temperature sensor. The temperature of the absorber layer was determined from the detailed balance of the experimental NIS spectra measured in the range of −20 to +50 meV (Figure S2, Supporting Information). The Sn-PDOS of sample S1 at 37 K is shown in Figure S3 (Supporting Information). Raman spectra of samples S1–S3 (Figure S4, Supporting Information) were also measured before device fabrication and are discussed in view of the composition and potential defects formation in the Supporting Information.

## 3 Results and Discussion

### 3.1 Sn-PDOS from NIS Measurements in the Dark

**Figure 1:**
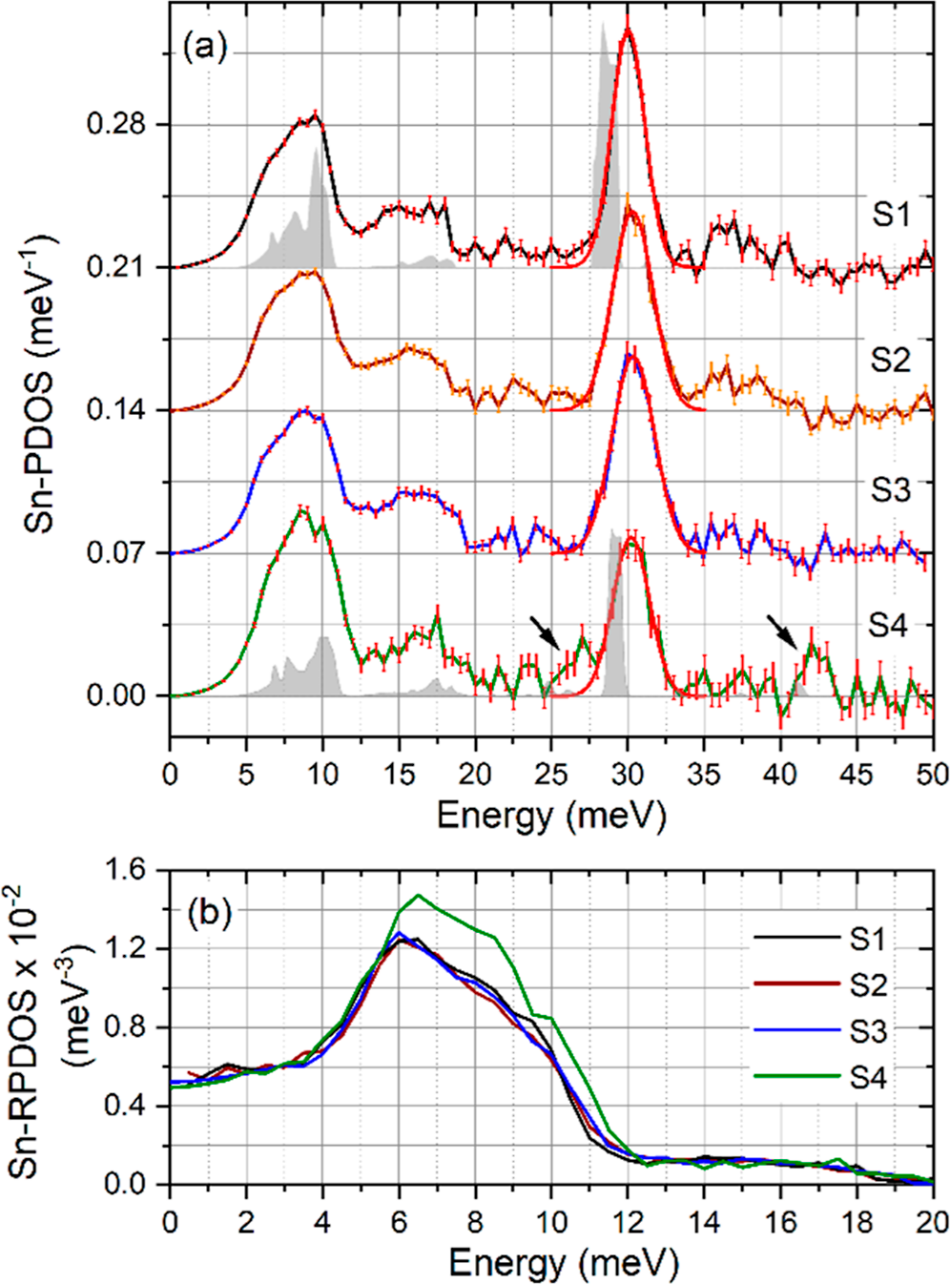
(a) Sn-PDOS and (b) reduced Sn-PDOS of the investigated samples. The curves in (a) are up-shifted by 0.07 meV^–1^ for the sake of clarity. The red solid line depicts a fit of the optical mode at ∼30 meV with a Gaussian (fit parameters are summarized in Table 2). The black arrows in (a) point to additional peaks at ∼27 and ∼42.5 meV in the S-containing sample. The calculated Sn-PDOS of the kesterite crystal structures of Cu_2_ZnSnSe_4_ and Cu_2_ZnSn(S_0.25,_Se_0.75_)_4_ are shown as gray shaded areas under the experimental Sn-PDOS of S1 and S4, respectively. The calculated Sn-PDOS data is reproduced from ref with permission from AIP Publishing.

The derived Sn-PDOS of the investigated samples are plotted in Figure 1a. First, comparing the S-free samples with near-(sample S1) and off-stoichiometric composition (samples S2 and S3), we find similar Sn-PDOS features: two broad peaks at approximately 9 and 16 meV, the main peak at 30 meV, as well as minor states between 20–25 and 34–42 meV. However, a careful inspection shows distinct differences. In particular, the exact position, full width at half-maximum (FWHM), and area of the peak at 30 meV vary, and the number of states (i.e., area under the PDOS curve) between 34 and 42 meV is smaller for S3 than for S1 and S2. To quantify the former effect, the peak at 30 meV was approximated by a Gaussian fit, shown by the red solid lines in Figure 1a, and the corresponding parameters are summarized in Table 2. For samples S2 and S3, the peak position shifts to higher energy by 1.0 and 1.1% (i.e., by 0.29 and 0.33 meV, respectively), whereas the FWHM increases by 24 and 32%, respectively, compared to sample S1. The peak areas of samples S1 and S2 remain the same, while for sample S3, an increase of 6.3% is found. This observation is in agreement with a broadening of the A2 and 230–250 cm^–1^ Raman peaks for off-stoichiometric composition (Figure S4, right panel, Supporting Information) and is attributed to the presence of certain lattice defect types in samples S2 and S3.^45−48^

**Table 2:** Energy, FWHM, and Area of the Peak at ∼30 meV, as Derived from the Gaussian Fits in Figure 1a

sample	energy (meV)	FWHM (meV)	area
S1	30.04(2)	2.5(1)	0.32(1)
S2	30.33(2)	3.1(1)	0.32(1)
S3	30.37(2)	3.3(1)	0.34(1)
S4	30.23(2)	2.9(1)	0.23(1)

To understand the experimentally derived Sn-PDOS of sample S1, it is compared with the *ab initio*-calculated Sn-PDOS^32^ of CZTSe with a kesterite structure (gray areas in Figure 1a, top). This comparison unveils that the main features discussed above are reproduced by theory, except for the phonon states between 34 and 42 meV, which are not present in the theoretically obtained Sn-PDOS.

The Sn-PDOS of the S-containing Cu_2_ZnSn(S_0.25_,Se_0.75_)_4_ absorber (sample S4, Figure 1a, bottom) shows overall similar main features to S1–S3 but with an increased (decreased) number of states around 9 (30) meV, additional peaks at 27 and 42.5 meV (indicated by black arrows in Figure 1a), and a reduced number of phonon states between 34 and 42 meV. Remarkably, the two additional peaks are predicted by theory^32^ (small gray-area peaks in Figure 1a bottom), with weak contributions at the lower end of the energy range of these features. The calculations^32^ suggest that they originate from a coupling of Sn atoms to the vibrations of Se and S atoms.

The experimentally observed phonon states around 9 and 16 meV appear approximately at the theoretically predicted positions, whereas the peak at 30 meV is shifted by ca. 1 meV to higher energy. This shift could be attributed to a deviation of the theoretically obtained lattice constants^32,49^ from the experimental values.^50^ In addition to the finite instrumental resolution, the broadening of the experimental Sn-PDOS arises from lattice defects, such as vacancies, interstitials, atomic substitutions, internal surfaces, and grain boundaries, which exist in polycrystalline thin-film solar cells. These effects are not taken into account by the calculations performed for a stoichiometric composition and a perfect kesterite crystal structure.^32^

To compare the low-energy part of the Sn-PDOS (arising primarily from the acoustic phonon modes^51^), it is convenient to plot the reduced PDOS^52^ (RPDOS = PDOS × *E*^–2^) of all samples in the energy range of 0–20 meV (Figure 1b). This plot shows that, while samples S1–S3 are very similar, sample S4 exhibits an increased number of phonon states in the energy range of 6–12 meV. The calculated phonon dispersions^32^ reveal that, in addition to the acoustic branches, a small number of low-energy optical branches contribute to the PDOS between 6 and 12 meV. Their number is higher for the S-containing absorber, which leads to the experimentally observed difference between S1–S3 and S4 in this energy range.

Furthermore, we discuss the experimentally observed phonon states around 16 meV and in the ranges of 20–25 and 34–42 meV, which are not present in the calculated Sn–PDOS^32^ (see the gray Sn–PDOS at the top of Figure 1a). A careful inspection of the *ab initio*-calculated Se-PDOS shows that the optical phonon states appear between 20 and 29 meV.^32^ Since Sn is directly bonded to Se, one might speculate that the observed phonon states in the Sn-PDOS between 20 and 25 meV originate from vibrations of Sn coupled to those of Se atoms. In contrast, the phonon states around 14 meV and in the range 34–42 meV in the Sn-PDOS of CZTSe cannot be attributed to similar coupled vibrational modes. To clarify the origin of these phonon states, the Sn-PDOS of sample S1 was also obtained at 37 K. The data (Figure S3, Supporting Information) show a significant reduction in the number of phonon states around 14 and between 34 and 42 meV. This result implies, in agreement with the *ab initio* calculations, that these phonon states do not belong to the Sn-PDOS of the CZTSe kesterite. Instead, they originate from multiphonon excitations,^53^ which are suppressed at lower temperatures and not fully eliminated by the data reduction algorithm^44^ employed for the 295 K spectrum. This, however, does not influence our conclusions since we compare the relative changes in the Sn-PDOS of the investigated samples obtained at room temperature.

### 3.2 Investigation of Defect Types and Secondary Phases

**Figure 2:**
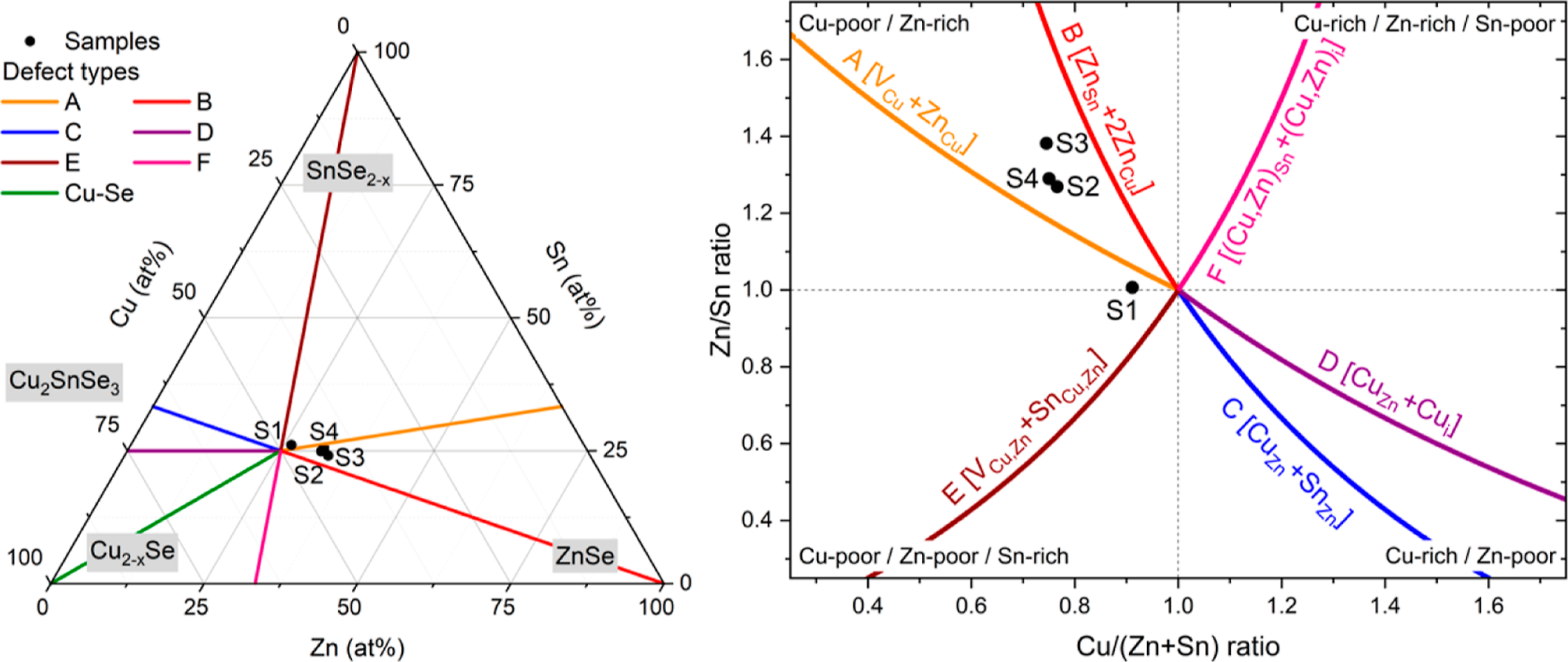
Ternary composition triangle with the possible secondary phases (left) and a cation ratio plot (right) with possible defect types^48,54−57^ for samples S1–S4 (solid black circles).

Combined theoretical and experimental studies demonstrated that, in kesterite samples with controlled compositions, only specific defect types and secondary phases can be formed.^26,48,54−57^ Moreover, a detailed investigation of the solar-cell efficiencies (up to 11%, close to that of sample S3) for various absorber compositions was performed to understand the impact of the defect types.^26,58,59^Figure 2 (left) shows a ternary composition diagram depicting the Cu–Zn–Sn contents as determined by EDX (Table 1) in atomic percentages, and Figure 2 (right) plots the cation ratio with the possible defect types depending on the composition.^26,48,54−57,60^ Samples S2, S3, and S4 lie in the Cu-poor, Zn-rich region (top left quadrant in the right panel of Figure 2), mainly forming defect types A (V_Cu_ + Zn_Cu_) and B (Zn_Sn_ + 2Zn_Cu_). Based on the composition of these samples, the formation of a ZnSe secondary phase^61−64^ is expected (left panel in Figure 2). For samples S1, A, and E (V_Cu,Zn_ + Sn_Cu,Zn_), defect types can be expected. However, all other defect types are also likely to occur, due to the almost stoichiometric composition. These defect types are unfavorable for well-performing solar cells since some of the defects are deep within the band gap and can act as strong recombination centers.^4,26,35,42−45,59,65^ Besides, sample S1 may also contain other binary (ZnSe, SnSe_2–*x*_, Cu_2–*x*_Se) and ternary (Cu_2_SnSe_3_) phases^48,54−57,60^ (see the left panel in Figure 2).

Consequently, sample S1 (i.e., the device with the lowest efficiency) might have a multitude of defect types and secondary phases. For example, Sn_Zn_ and Cu_Zn_ + Sn_Zn_ are expected to form deep carrier trap states.^9,26,59,65^ The Cu_Zn_ + Sn_Zn_ defect type could be prevalent since the attractive interaction between the two defects could lead to a lower formation energy.^66^ In addition, Sn_Zn_^+2^ and [Cu_Zn_ + Sn_Zn_]^+^ exhibit large carrier-capture cross sections, which can lead to an enhancement of the nonradiative recombination and thus reduce the PCE.^36^

Furthermore, previous studies demonstrated that the defect types can affect the frequency, shape, and intensity of Raman-active modes.^26,58,67,68^ The mode intensity is proportional to the number of phonon states at the Γ point (center of the Brillouin zone) of respective elements and relates to the concentration of vibrating ions.^26,34,58^ The Raman spectrum of sample S3 (Figure S4, left panel, Supporting Information) shows a reduced intensity at 173 cm^–1^ (i.e., the A2 Raman mode), compared to those of S1 and S2. This mode mainly relates to Cu vibrations and corresponds to increased Cu vacancies (*V*_Cu_, decreased Cu content) and the presence of Zn_Cu_ antisite defects (A-type).^26,58,67^ A Cu-poor and Zn-rich composition favors the formation of Zn_Cu_ (increased Zn content) defects.^58^ It was reported^26,58,67^ that the presence of Zn_Cu_ and Zn_Sn_ antisite defects (B-type) could give rise to intensity variations between 230 and 250 cm^–1^ (Figure S4, inset left panel, Supporting Information). This observation indicates a Cu-poor and Zn-rich composition with dominating defect types A and B in sample S3, compared to S1 and S2, as discussed above (upper left quadrant in Figure 2, right panel).

The fact that the PCE remains below the theoretically predicted values indicates that other defects could be present in the bulk of the light-absorbing CZTSe layer, which is not accessible by Raman spectroscopy with its characteristic 1/*e* attenuation length of 50–60 nm but is probed by the NIS experiment. It has recently been reported that the increased Cu–Zn disorder facilitates the formation of Sn_Zn_-related defects, which are detrimental to the *V*_OC_.^69^ These defects cause a lattice distortion and shortening of the Sn–Se bond length, leading to a shift of the optical modes to higher energy.^36,37,70,71^ We speculate that Sn_Zn_-related defects, along with the A and B defect types discussed above, lead to the observed slight shift of the main peak at 30 meV to higher energy, as well as to an increase of its FWHM in the Sn-PDOS from S1 to S3 (Table 2).

**Table 3:** Energy, FWHM, and Area of the Peak at ∼30 meV, as Derived from the Gaussian Fits in Figure 3a

S3	energy (meV)	FWHM (meV)	area
before	30.37(2)	3.3(1)	0.34(1)
*operando*	30.26(3)	3.3(1)	0.31(1)
open-circuit	30.06(2)	3.3(1)	0.32(1)
after	30.15(4)	3.5(1)	0.33(1)

### 3.3 Sn-PDOS from NIS Measurements under Illumination

**Figure 3:**
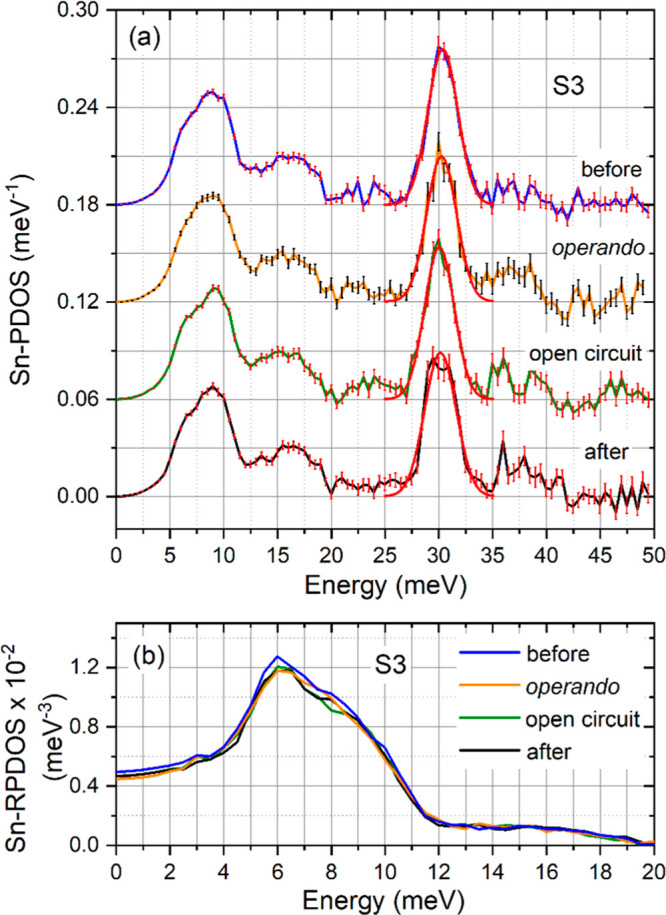
(a) Sn-PDOS and (b) reduced Sn-PDOS of the sample S3 obtained under the given experimental conditions. The curves in (a) are up-shifted by 0.06 meV^–1^ for clarity. The red solid line depicts the Gaussian fit of the optical mode at ∼30 meV. The obtained parameters are summarized in Table 3. The variation in the number of phonon states between 34 and 42 meV originates from multiphonon excitations (see the Supporting Information).

Figure 3a shows the Sn-PDOS of S3 obtained under illuminated “*operando*” conditions (i.e., at the maximum power point, MPP) and in “open-circuit” mode, in comparison with the Sn-PDOS obtained under “dark” conditions “before” and “after” the “*operando*” measurements (see the Experimental Section and Supporting Information for a description of the experimental approach). Interestingly, in Sn-PDOS, a systematic change in the position and area of the main peak at 30 meV during the “*operando*”, “open-circuit”, and “after” measurements is visible. In order to quantify this effect, the peak is fitted by a Gaussian profile (red lines in Figure 3a), and the obtained parameters are summarized in Table 3. These values show that the peak position shifts gradually to lower energy by 0.7%, the FWHM increases by 6.1%, and the area is reduced by 2.9%. Similar to the Sn-PDOS of S1–S3 (Figure 1a), variations of the Sn-PDOS in the energy range of 34–42 meV arising from multiphonon excitations are visible (see the Supporting Information).

It was reported that, in alloys, a red shift (lattice softening) under light illumination (particularly visible in the Sn-PDOS at “open-circuit” mode) could be associated with an increased concentration of free charge carriers, leading to a screening between the light-generated charge carriers and the lattice ions.^72−74^ Furthermore, it was reported^75−77^ for Cu(In,Ga)Se_2_ (CIGSe) and other perovskite solar cells^75^ that light-soaking (blue, red, and white light illumination) and voltage bias can induce metastable defects that can trap charge carriers and lead to a lattice relaxation.^75−77^ The proposed metastable defects in CIGSe are In_Cu_ antisites, Cu interstitials, and Se vacancies in a Se–Cu divalent complex (*V*_Se_–*V*_Cu_).^76−79^ For CZTSSe, an applied voltage bias can induce Zn^2+^ and Se^2–^ ion migration.^80^ It was also deduced that, in CZTSe, about 3 h of light soaking could lead to low-energy Cu_Zn_ and V_Cu_ defects and a high-energy metastable defect complex (*V*_Se_–*V*_Cu_).^81^ A variation in the metastable defects was studied before and after AM1.5 illumination and applied bias, respectively. It was suggested that high-energy defect states, which could trap the charge carriers, are formed in the absorber during the illumination.^82,83^ The metastable defects could be removed only upon heat treatment at high temperatures.^83^ The open-circuit voltage after the NIS experiment remained unchanged compared with the starting value, suggesting that the general quality of the device did not degrade.

Based on these findings, we conclude that the observed changes in the Sn-PDOS of sample S3 (Figure 3a and Table 3) after the 40 h exposure to visible light, X-rays, and applied bias originate from the formation and migration of metastable defects and/or a partial structural relaxation. The reduced Sn-PDOS (Figure 3b) demonstrates that the acoustic and low-energy optical phonon states remain unaffected by light irradiation and device operation.

### 3.4 Thermodynamic and Elastic Properties

The calculated^84^ thermodynamic (vibrational entropy *S*_v_ and lattice specific heat capacity *C*_v_) and elastic (mean square atomic displacement <*x*^2^> and mean force constant *F*) properties from the experimental Sn-PDOS are summarized in Table 4. A clear trend in these parameters from samples S1 to S3 cannot be identified, except for the force constant, which reaches its lowest value in S3. This behavior, as well as the increase in <*x*^2^> and *S*_v_, can be explained by the change from a near-stoichiometric composition in S1 to an off-stoichiometric composition in S3. At *operando* and open-circuit conditions, <*x*^2^> increases by 2.2 and 4.3% (respectively), *F* by 8.3 and 6.7% (respectively), and *S*_v_ by 2.7% compared to the dark measurement of S3. The values of <*x*^2^> and *F* derived at dark conditions “after” the *operando* measurements deviate from the corresponding “before” values by −5.4 and +11.0%, respectively. Tentatively, this effect might be interpreted as a formation and migration of metastable defects and/or relaxation of the crystal lattice during light irradiation.

**Table 4:** Temperature *T*, Mean Square Atomic Displacement <*x*^2^>, Mean Force Constant *F*, Vibrational Entropy *S*_v_, and Lattice Specific Heat Capacity at Constant Volume *C*_v_, Calculated from the Sn-PDOS of the Investigated Samples under the Given Conditions

sample	*T* (*K*)	<*x*^2^> (Å^2^)	*F* (Nm^–1^)	*S*_v_ [3 *k*_B_]	*C*_v_ [3 *k*_B_]
S1	295(1)	0.0094(3)	235(6)	2.22(2)	0.96(1)
S2	295(1)	0.0092(3)	237(6)	2.20(2)	0.95(1)
S3-before	295(1)	0.0092(3)	218(6)	2.20(1)	0.94(1)
S3-*operando*	317(7)	0.0094(3)	236(6)	2.25(2)	0.95(1)
S3-open-circuit	326(6)	0.0096(3)	233(6)	2.27(2)	0.95(1)
S3-after	295(1)	0.0087(3)	242(7)	2.19(2)	0.96(1)
S4	295(1)	0.0104(3)	210(8)	2.41(2)	1.00(1)

The addition of S to CZTSe causes a further softening of the lattice. Sample S4 is characterized by the highest <*x*^2^>, *S*_v_, and *C*_v_ (compared to the S1–S3 samples), and an *F* value comparable to S3 (before the light irradiation). This is a consequence not only of the presence of additional phonon states at 27 and 42.5 meV but mostly also of the enhanced number of phonon states at the low-energy part of the Sn-PDOS (Figure 1b).

From the detailed balance of the phonon annihilation and creation part of the NIS spectrum, the temperature of the light-absorbing layer during solar cell operation can be calculated (Figure S2, Supporting Information). The derived temperatures at *operando* and open-circuit conditions, displayed in Table 4, show an increase of 22 and 31 K, respectively, compared to the ambient temperature (295 K). The independently measured temperatures of the solar-cell surface during the *operando* [308(1) K] and open-circuit [306(1) K] experiments are lower by 9 and 20 K, respectively, than the temperature of the CZTSe absorber. This result agrees with the reported difference of approximately 10 K between the solar-cell interior and its surface, which is attributed to the difference between the light-absorption coefficients, specific heat capacities, and thermal conductivities of the top glass layer and the light-absorbing material.^85^

## 4 Conclusions

In summary, we have determined the Sn-PDOS of Cu_2_ZnSnSe_4_ kesterite thin-film solar cells, with PCEs of 3.2, 7.6, and 10.6%, using nuclear inelastic X-ray scattering on the Mössbauer-active isotope ^119^Sn. Although the Sn-PDOS of the three devices are similar, they exhibit distinct differences. Specifically, a systematic shift to higher energies and broadening of the most prominent peak at 30 meV are clearly detected, which is correlated with the type and concentration of defects characteristic for the investigated compositions. The Sn-PDOS of the device with the highest efficiency was additionally determined at “*operando*” and “open-circuit” conditions. The Sn-PDOS unveils a systematic red shift (lattice softening) of the main optical phonon peak at 30 meV. A comparison of the data obtained under “dark” conditions before and after the “*operando*” and “open-circuit” measurements demonstrates that these variations are irreversible and suggests that structural changes have taken place. These changes are attributed to metastable defects formation, migration, and/or lattice relaxation processes as a consequence of the extended light-soaking, X-ray exposure, and/or applied bias-voltage during the solar cell operation. The temperature of the CZTSe absorber at “*operando*” and “open-circuit” conditions was estimated from the detailed balance of the NIS spectra and was found to be higher by 9 and 20 K, respectively, than the temperature measured on the device surface. Additionally, the Sn-PDOS of the Cu_2_ZnSn(S_0.25_Se_0.75_)_4_ absorber was determined and found to exhibit features close to those of CZTSe, with additional phonon states at 27 and 42.5 meV arising from a coupling of Sn atoms to the vibrations of Se and S atoms. The experimentally obtained Sn-PDOS are in qualitative agreement with the DFT-calculated Sn-PDOS of CZTSe and CZTSSe with a kesterite structure and allow for a comprehensive determination of the lattice dynamics, thermodynamics, and elastic properties of these important materials. Furthermore, this work demonstrates that advanced lattice dynamics studies, including under *operando* conditions, might contribute toward further improvement of the efficiency of kesterite-based and other thin-film solar cells for PV and PEC.

## Data Availability

The data that support the findings of this study are available from the corresponding authors upon reasonable request.

## Supporting Information Available

Details of the NIS experimental setup, temperature calculation from the NIS measurements, NIS experiment at 37 K, and Raman measurements and analysis (PDF)
