# Supplementary material for: Lattice Dynamics of Cu2ZnSn(Sx,Se1–x)4 Kesterite Thin-Film Solar Cells Studied by Nuclear Inelastic Scattering
Source: J Phys Chem C Nanomater Interfaces. 2024 Oct 7;128(41):17483–91. doi: 10.1021/acs.jpcc.4c03689 (PMC11493056; doi:10.1021/acs.jpcc.4c03689)
Supplement: Supplementary file 1 — jp4c03689_si_001.pdf [file jp4c03689_si_001.pdf]

Supporting Information

Lattice Dynamics of $Cu_2ZnSn(S_x,Se_{1-x})_4$ Kesterite Thin-Film Solar Cells Studied by Nuclear Inelastic Scattering

Raju Edla,[,†], David Nowak[‡], Dirk Hauschild[†,§,ǀ], Ilya Sergueev[⊥], Devendra Pareek[‡], Levent Gütay[‡], Clemens Heske[†,§,ǀ], Lothar Weinhardt[†,§,ǀ], and Svetoslav Stankov[*,†,#]*

[†]Institute for Photon Science and Synchrotron Radiation (IPS), Karlsruhe Institute of Technology (KIT), Karlsruhe 76131, Germany

[‡]Ultrafast Nanoscale Dynamics, Institute of Physics, Carl von Ossietzky University of Oldenburg, Oldenburg 114-118-26129, Germany

[§]Institute for Chemical Technology and Polymer Chemistry (ITCP), Karlsruhe Institute of Technology (KIT), Karlsruhe 76131, Germany

[ǀ]Department of Chemistry and Biochemistry, University of Nevada, Las Vegas (UNLV), Las Vegas, Nevada NV 89154, United States.

[⊥]Deutsches Elektronen-Synchrotron DESY, Hamburg 22607, Germany

[#]Laboratory for Applications of Synchrotron Radiation (LAS), Karlsruhe Institute of Technology (KIT), Karlsruhe 76131, Germany

*** Authors to whom correspondence should be addressed:**

edla.raju@kit.edu, svetoslav.stankov@kit.edu

Details of the NIS experimental setup

Figure S1 shows photographs of the setup for nuclear inelastic scattering (NIS) measurements under dark (left picture) and light irradiation (right picture) conditions, including the kesterite solar cell, the avalanche photodiode (APD) detector, and the optical light guides. During the dark measurements, the distance between the solar cell and the APD was approximately 1.0 mm, ensuring a large solid angle for detecting the fluorescent x-rays. For the light irradiation (*operando*) measurements, the distance between the sample and the APD was approximately 10 mm to ensure an efficient illumination of the solar cell. This resulted in a decrease in the count rate in the experiment by about a factor of three.

The solar cell (sample S3, see main paper) measured under *operando* conditions has a power conversion efficiency of 10.6 % and consists of a Molybdenum (Mo) back contact, the $Cu_2ZnSnSe_4$ (CZTSe) solar absorber (~2 μm thickness), a 50 nm CdS buffer layer, and a transparent window layer (i-ZnO and Al:ZnO) as electrical front contact. Electrical point contacts were made from Cu wires, fixed on the Mo and ZnO layers with silver paint (PLANO GmbH). The NIS experiments were performed under the following conditions: 1) in the dark (set-up as in Figure S1, left, light in the experimental hutch was turned off); 2) during solar-cell operation at maximum power point (MPP) under visible-light illumination ("*operando*", Figure S1, right); 3) in open-circuit mode under visible-light illumination (Figure S1, right). The MPP was selected from an I-V curve using a Biologic SP-240 Potentiostat with "EC-Lab^{TM}" software. The open-circuit voltage (V_{OC}) was measured to be about 450 mV. For light irradiation onto the solar cell, two PHILIPS halogen cold light sources (150 W, 3400 K) were used, equipped with one and two glass fiber optical light guides, respectively.

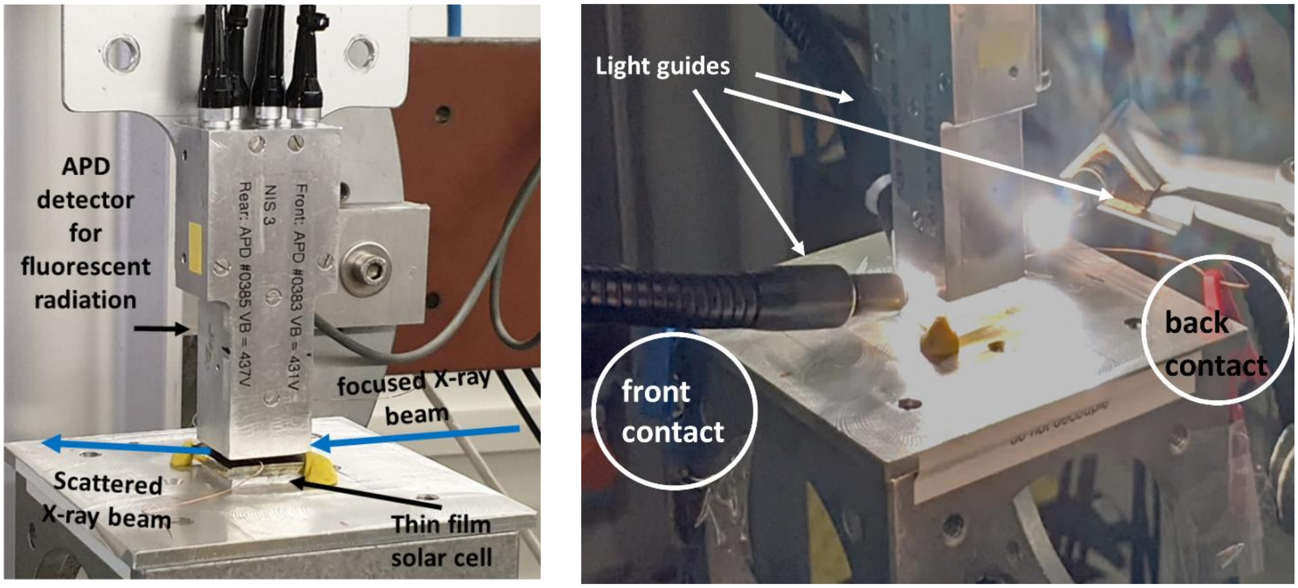

Figure S1. The setup for NIS experiments under dark (left) and visible-light illumination conditions (right) at the beamline P01 of PETRA III.

Temperature calculation from the NIS measurements

Figure S2 shows the nuclear inelastic absorption spectra of sample S3, obtained at the indicated conditions. The central line at 0 meV energy transfer corresponds to the resonant recoil-free (elastic) absorption of the x-rays with an energy of 23.88 keV by the nuclei of ^{119}Sn. The sidebands in the spectrum correspond to the nuclear resonant absorption associated with an energy transfer from the x-rays to the lattice (phonon creation, E > 0) and from the lattice to the x-rays (phonon annihilation, E < 0). Both sides of the spectrum *S(E)* obey the detailed balance equation: $S(E) = e^{-\beta E} S(-E)$, where $\beta = 1/k_B T$, with k_B being the Boltzmann constant and T the temperature. By comparing the left- and right-hand sides of the NIS spectrum in a given energy interval, the temperature of the sample can be calculated from the detailed balance. This approach was used to estimate the temperature of the CZTSe light absorber during the *operando* and open-circuit NIS measurements of sample S3.

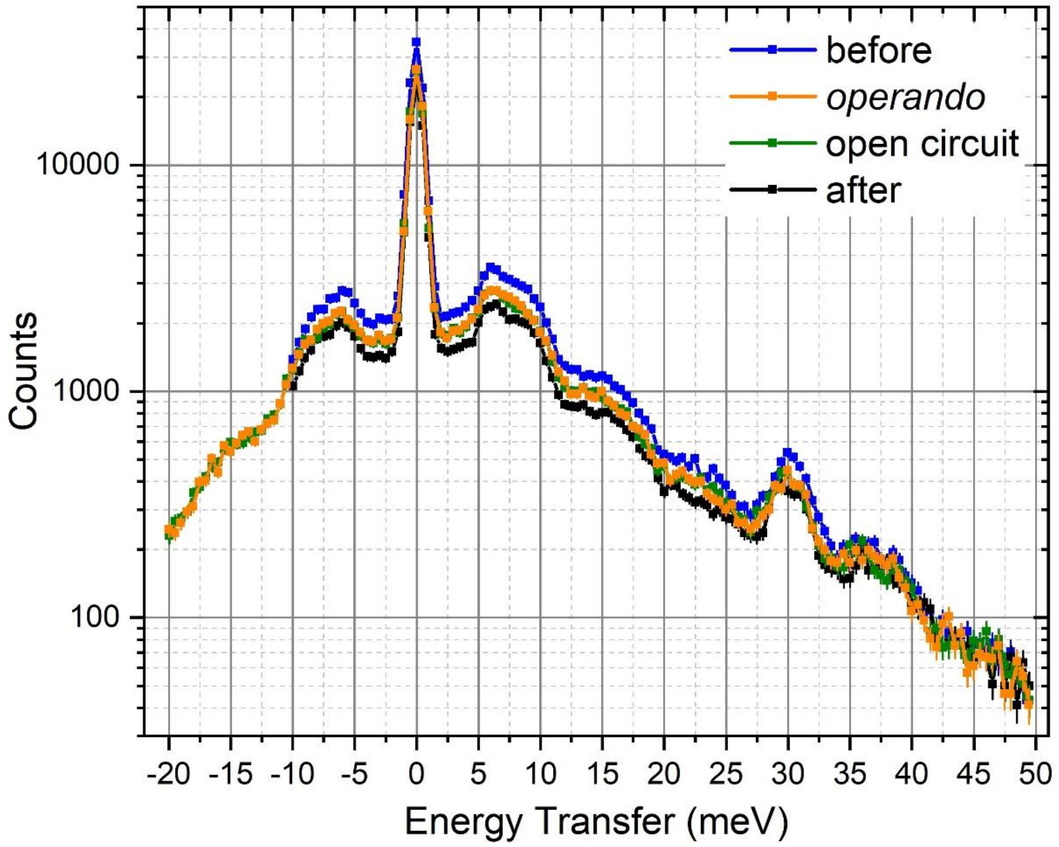

Figure S2. Nuclear inelastic absorption spectra of sample S3 obtained under the indicated conditions.

Low-temperature NIS experiment on sample S1

The determination of the PDOS from a NIS experiment relies on a separation of the single-phonon from multi-phonons excitation/annihilation events. The mathematical procedure for separating the one-phonon from multiphonon terms in the harmonic approximation is well established[1] and works very well for relatively high values of the probability for nuclear resonant absorption/emission of γ-rays given by the Lamb-Mössbauer factor f_{LM} (the lower f_{LM}, the higher the probability for multiphonon excitations). In the investigated samples, f_{LM} has moderate values at room temperature (for S1 f_{LM}=0.27 at 295K). This fact, combined with the relatively low statistics of the NIS spectra (all samples contain the natural abundance of the Mössbauer-active isotope ^{119}Sn of 8.6%), especially for the ones obtained under light irradiation with an increased sample-to-detector distance, might lead to an incomplete subtraction of the multiphonons from the room temperature NIS data.

To investigate the impact of the multiphonon excitations on the Sn-PDOS obtained at room temperature, an additional NIS experiment (instrumental resolution of FWHM = 0.7 meV) was performed on sample S1 at 37 K. The sample was mounted in a helium continuous-flow cryostat and illuminated with the x-ray beam at an incidence angle of ca. 10°. Figure S3 compares the Sn-PDOS of sample S1 obtained at 295 K and 37 K. This comparison reveals that the phonon states around 14 meV and between 34 – 42 meV in the Sn-PDOS at 295 K originate from multiphonon excitations, which cannot be entirely eliminated in the data reduction process due to the moderate value of the Lamb-Mössbauer factor (f_{LM} = 0.27 at 295 K, compared to f_{LM} = 0.77 at 37 K).

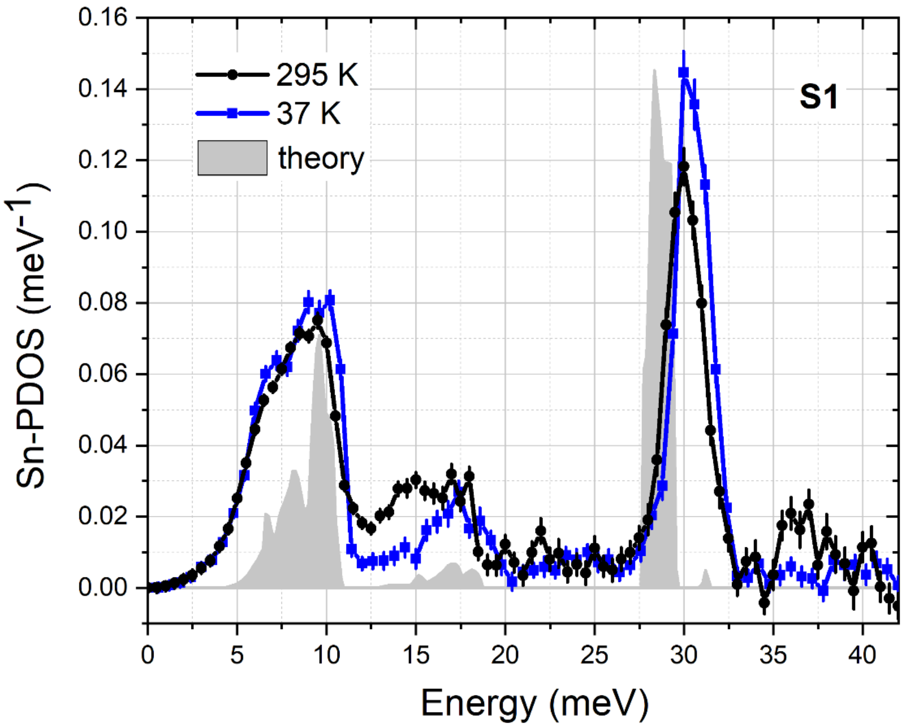

Figure S3. Sn-partial PDOS of sample S1 with PCE of 3.2 % at the given temperatures and the *ab initio*-calculated Sn-PDOS of CZTSe with kesterite structure (the theory data is reproduced from ref 2 with permission from AIP Publishing).

This, however, does not influence the drawn conclusions in the paper since we compare the relative changes in the Sn-PDOS of the investigated samples obtained at room temperature. Figure S3 demonstrates that all features in the *ab initio* Sn-PDOS (calculated at 0 K) of the CZTSe kesterite[2] can be

reproduced by the experimentally obtained Sn-PDOS of sample S1 at 37 K. By comparing the values of the mean force constant of S1 derived at 37 K (226 N/m) and at 295 K (235 N/m), which do not depend on temperature in the harmonic approximation[1], we estimate the error in the thermodynamic and elastic properties (Table 4 of the main paper) arising from the multiphonons contribution to the Sn-PDOS to be below 5%.

Raman experiment and data analysis

Raman spectroscopy was performed with a Horiba LabRAM Aramis setup equipped with a green laser (wavelength 532 nm, power 3 mW) and a spot size of ~1 μm at the sample position. The wavenumber axis was shifted to the central Rayleigh peak at 0 cm^{-1}, the detector background was subtracted, and then the spectra were normalized to the value at ~300 cm^{-1} (float background).

Raman measurements with the 532 nm laser source relate to a characteristic 1/*e* attenuation length[3] of 50 – 60 nm and are performed on the CZTSe absorber, i.e., without CdS and window layer. Accordingly, the information obtained is relatively surface-sensitive (as compared to the absorber thickness ~2 μm). In Figure S4, the spectra of samples S1-S3 show two intense Raman modes at 196 (A1) and 173 (A2) cm^{-1} and less intense features around 233 and 244 cm^{-1}. The peak at 173 cm^{-1} is dominated by Cu vibrations, with a noticeable contribution from Zn vibrations, whereas the peak at 196 cm^{-1} corresponds mainly to Se vibrations[4-6]. The peak at 234 cm^{-1} is dominated by Zn vibrations, and the broad peak around 244 cm^{-1} is sensitive to Sn vibrations, coupled with Zn atoms or, if present, with Zn_{Sn} anti-site defects in the disordered kesterite[4, 6, 7]. The changes in the Raman modes can be caused by the presence of defects, which can affect the mode frequency, shape, and intensity[6, 7]. The intensity of the modes is proportional to the number of phonons at the Γ-point of the Brillouin zone and relates to the concentration of vibrating ions in the probing volume[6, 7]. The black arrows indicate the variations in the intensity and broadening at A2 Raman mode induced by changes in the concentration and defects, respectively.

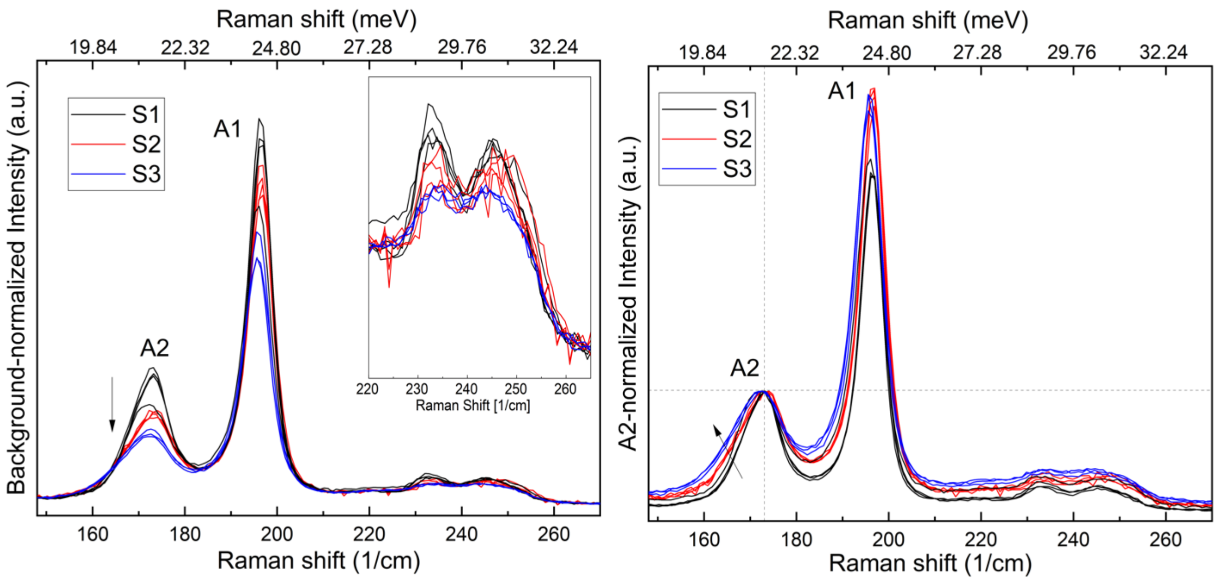

Figure S4. Raman spectra of samples S1, S2, and S3. The left figure shows the spectra normalized to their background at ~300 cm^{-1}, and the right figure shows the data normalized to the maximum of the A2 peak. The spectra were collected with a 532 nm laser at a power of 3 mW. Raman spectra plotted with color shades were collected on different locations to confirm the homogeneity of the samples.

References

(1) Kohn, V. G.; Chumakov, A. I. DOS: Evaluation of phonon density of states from nuclear resonant inelastic absorption. *Hyperfine Interact.* **2000**, *125* (1), 205-221. DOI: 10.1023/A:1012689705503.

(2) Khare, A.; Himmetoglu, B.; Cococcioni, M.; Aydil, E. S. First principles calculation of the electronic properties and lattice dynamics of $Cu_2ZnSn(S_{1-x}Se_x)_4$. *J. Appl. Phys.* **2012**, *111* (12), 123704. DOI: 10.1063/1.4728232.

(3) Taskesen, T.; Pareek, D.; Hauschild, D.; Haertel, A.; Weinhardt, L.; Yang, W.; Pfeifelmann, T.; Nowak, D.; Heske, C.; Gütay, L. Steep sulfur gradient in CZTSSe solar cells by H_2S-assisted rapid surface sulfurization. *RSC Adv.* **2021**, *11* (21), 12687-12695. DOI: 10.1039/D1RA00494H.

(4) Mortazavi Amiri, N. B.; Postnikov, A. Electronic structure and lattice dynamics in kesterite-type $Cu_2ZnSnSe_4$ from first-principles calculations. *Phys. Rev. B* **2010**, *82* (20), 205204. DOI: 10.1103/PhysRevB.82.205204.

(5) Dimitrievska, M.; Giraldo, S.; Pistor, P.; Saucedo, E.; Pérez-Rodríguez, A.; Izquierdo-Roca, V. Raman scattering analysis of the surface chemistry of kesterites: Impact of post-deposition annealing and Cu/Zn reordering on solar cell performance. *Sol. Energy Mater. Sol. Cells* **2016**, *157*, 462-467. DOI: 10.1016/j.solmat.2016.07.009.

(6) Dimitrievska, M.; Oliva, F.; Guc, M.; Giraldo, S.; Saucedo, E.; Pérez-Rodríguez, A.; Izquierdo-Roca, V. Defect characterisation in $Cu_2ZnSnSe_4$ kesterites via resonance Raman spectroscopy and the impact on optoelectronic solar cell properties. *J. Mater. Chem. A* **2019**, *7* (21), 13293-13304. DOI: 10.1039/C9TA03625C.

(7) Dimitrievska, M.; Fairbrother, A.; Saucedo, E.; Pérez-Rodríguez, A.; Izquierdo-Roca, V. Influence of compositionally induced defects on the vibrational properties of device grade $Cu_2ZnSnSe_4$ absorbers for kesterite based solar cells. *Appl. Phys. Lett.* **2015**, *106* (7), 073903. DOI: 10.1063/1.4913262.
